# Phase 2 trial of intravenous oncolytic virus JX-594 combined with low-dose cyclophosphamide in patients with advanced breast cancer

**DOI:** 10.1186/s40164-022-00338-2

**Published:** 2022-12-06

**Authors:** Sophie Cousin, Maud Toulmonde, Michèle Kind, Jean-Philippe Guegan, Alban Bessede, Coralie Cantarel, Carine Bellera, Antoine Italiano

**Affiliations:** 1 Department of Medical Oncology, Early Phase Trials and Sarcoma Units, Institut Bergonié, 229 Cours de l’Argonne, Bordeaux, France; 2 Department of Medical Imaging, Institut Bergonié, Bordeaux, France; 3 Explicyte, Bordeaux, France; 4 Bordeaux Population Health Research Center, Univ. Bordeaux, Epicene team, UMR 1219, F-33000 Inserm, Bordeaux, France; 5 Clinical and Epidemiological Research Unit, Comprehensive Cancer Center, Inserm CIC1401, Institut Bergonié, F-33000 Bordeaux, France; 6 Faculty of Medicine, University of Bordeaux, Bordeaux, France

**Keywords:** Breast cancer, Oncolytic virus, JX-594

## Abstract

Breast cancer is one the most common cause of cancer death in women worldwide. We report here the first phase II study investigating a virus genetically engineered for tumor-selective replication in patients with breast cancer. Ten patients were treated with a combination of low-dose oral cyclophosphamide and intra-venous JX-594, a thymidine kinase gene-inactivated oncolytic vaccinia virus engineered for the expression of transgenes encoding human granulocyte-macrophage colony-stimulating factor (GM-CSF) and β-galactosidase. Best response as per RECIST criteria was stable disease for 2 patients and progressive disease for 8 patients. Median progression-free and overall survival were 1.6 months (95% CI: [1.1–1.9]) and 14.4 months (95% CI: [2.0 – NA]) respectively. High throughput analysis of sequential plasma samples revealed an upregulation of protein biomarkers reflecting immune induction such as IFN gamma. Whether the combination of JX-594 with an immune checkpoint inhibitor is associated with meaningful clinical activity is therefore worth to investigate.

**Supplementary Information:**

The online version contains supplementary material available at 10.1186/s40164-022-00338-2.

To the Editor

Breast cancer is one the most common cause of cancer death in women worldwide. Patients with metastatic hormone receptor positive breast cancer can be eligible for several lines of hormonal therapy, targeted therapy, and chemotherapy. However, whatever the treatment used, secondary resistance is the rule with subsequent disease progression. Additional therapeutic options are therefore needed to improve overall survival.

Oncolytic viruses (OV) represent a new therapeutic approach for the management of cancer with specific features compared with conventional anti-cancer agents. JX-594 (pexastimogene devacirepvec; PEXA-VEC) is a thymidine kinase gene-inactivated oncolytic vaccinia virus engineered for the expression of transgenes encoding human granulocyte-macrophage colony-stimulating factor (GM-CSF) and β-galactosidase [1]. In phase 1/2 trials of intratumoral injection into liver cancers [2, 3], JX-594 had an excellent safety profile and was associated with tumor shrinkage and a dose-related clinical benefit.

However, for patients with refractory, widespread metastatic tumors, intravenous administration of a high dose of JX-594 may be required to achieve systemic oncolytic tumor responses. Such an approach has been evaluated in a phase 1 trial which included 23 patients with advanced solid tumors and who received a single intravenous infusion of JX-594 [4]. The results showed a dose-related antitumor activity was observed and the lack of toxicity on normal tissues.

We report here the safety and efficacy of efficacy of JX-594 combined with metronomic chemotherapy in patients with hormone receptor positive (HR+) breast cancer.

This was a single-arm, phase 2 clinical trial based on an optimal Simon’s two-stage design [5]. Patients received 50 mg of cyclophosphamide (CP) orally b.i.d. one week on and one week off and JX-594 at the dose 1.10^9^ plaque forming units (pfu) every 2 weeks for the first 3 injections and then every 3 weeks until disease progression and/or unacceptable toxicity. Details regarding eligibility criteria, design and statistical analysis are provided as supplementary data (online).

Between May 11th, 2017 and April 4th, 2018, 10 patients with advanced breast hormone receptor positive HER2 negative breast cancer were enrolled at Institut Bergonié (Bordeaux, France). Baseline patient characteristics are listed in Table 1. After a median follow-up of 10.9 months (95% CI 1.6–25.6), all the patients discontinued treatment. Discontinuation was related to disease progression in all the cases. Best response as per RECIST criteria was stable disease for 2 patients and progressive disease for 8 patients (Supplementary Fig. 1). Median progression-free and overall survival were 1.6 months (95% CI: [1.1–1.9]) and 14.4 months (95% CI: [2.0 – NA]) respectively (Supplementary Fig. 2).

**Table 1:** Patient characteristics (n = 10)

**Gender, n (%)**	
Male	0 (0)
Female	10 (100)
**Age**	
Median, years (range)	61 (34–70)
**ECOG PS, n (%)**	
0	6 (60)
1	4 (40)
**Histological subtype, n (%)**	
Ductal carcinoma	8 (80)
Lobular carcinoma	2 (20)
**Liver metastases**	
Yes	6 (60)
No	4 (40)
**Stage, n (%)**	
Locally advanced	1 (10)
Metastatic	9 (90)
**Prior line(s) of chemotherapy, n (%)**	
1	1 (10)
2	1 (10)
> 2	8 (80)

All 10 patients were included in the safety analysis. At the time of analysis, 25 cycles of JX-594 and metronomic CP had been administered, with a median of two cycles per patient (range 1–6). The most observed toxicity was fever which occurred for all (100%) patients (supplementary Table 1). Grade 3 toxicities were observed in four patients and included one case of grade 3 fever and one case of grade 3 lymphopenia (supplementary Table 1). No grade 4 toxicity was observed.

To evaluate the immune response to oncolytic virotherapy, we performed a proteomic analysis of plasma samples as previously described [6]. Although only few changes in plasma proteome were observed between C1D8 and baseline (first administration of low dose cyclophosphamide), comparison of plasma samples between C1D22 and C1D8 (first injection of JX-594) revealed a significant upregulation of several proteins which reflect immune induction such as CD8A, CD6 (a lymphocyte surface co-receptor physically associated with the T-cell receptor), CCL19 ( a critical regulator of the induction of T cell activation),IFN gamma as well as Tnfrsf9 (4-1BB) (Fig. 1).

**Fig. 1:**
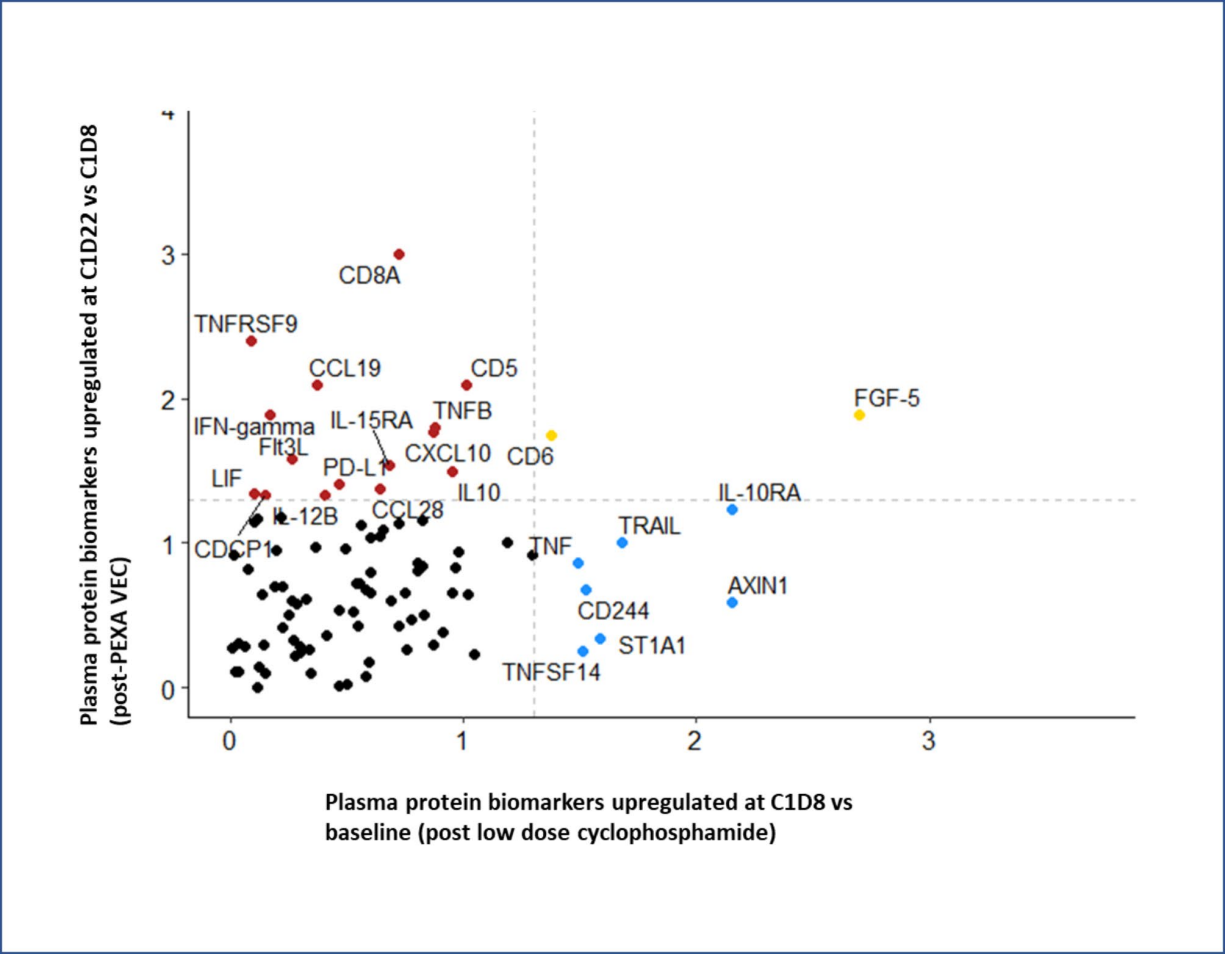
Plasma protein biomarkers upregulated after administration of low-dose cyclophosphamide (x axis) or JX-594 (y axis)

The oncolytic viruses currently developed for the treatment of patients with advanced cancer can be classified into three categories (i) wildtype mutant viruses, (ii) viruses genetically modified to replicate selectively in tumor cells and (iii) viruses genetically engineered to boost the immune system.

Most of the data related to the safety and efficacy of oncolytic viruses in patients with advanced metastatic breast cancer come from early phase study including patients with advanced tumors of which only a few were breast cancers [7]. As observed, in our study, the results of these studies show that treatment with oncolytic viruses were well tolerated with few adverse events mostly limited to flu-like symptoms such as fever. The safety profile observed in our study was similar to that observed in previous study investigating systemic administration of JX-594 in patients with other solid tumor types such as colorectal cancer [4, 8].

Only one Phase II trial designed to assess the efficacy of an oncolytic virus in patients with breast cancer was reported. This study evaluated the safety and efficacy of the reovirus pelareorep administered intravenously in combination with paclitaxel [9]. This study did not reach its first endpoint of progression-free survival.

We report here the first phase II study investigating a virus genetically engineered for tumor-selective replication in patients with breast cancer. Although, we observed low clinical activity, the results of our study pave the way for innovative approach to be evaluated in hormone receptors breast cancers.

Immune checkpoint blockade has been shown to have modest activity in this group of tumors which are poorly infiltrated by immune cells and have low expression of PD-L1 on their surface [10]. By activating an immune response to the tumor cells due to viral infection, OVs have the potential to turn the “cold” tumor microenvironment to “hot” and to sensitize tumors to immune checkpoint inhibitors as recently demonstrated in a pre-clinical model of triple negative breast cancer (11–12). Interestingly, by using plasma proteomics, we have found that JX-594 induced a strong anti-viral immune response accompanied by the production of cytokines such as type-1 interferons and chemokines playing a key role in lymphocytic activation (Fig. 1). Interestingly, it has been shown that targeting Tfrsf9 – induced upon JX-594 – in combination with an oncolytic virus is able to induce an anti-tumor immune response that translated into tumor growth delay in a murine preclinical model [13]. Whether the combination of JX-594 with an immune checkpoint inhibitor is associated with meaningful clinical activity is therefore worth to investigate. A clinical trial evaluating the combination of JX-594 in combination with Avelumab in patients with advanced breast cancer is ongoing (NCT02630368).

## Electronic supplementary material

Below is the link to the electronic supplementary material.

Supplementary Material 1: Supplementary Methods and Results

## Data Availability

Not applicable.

## Declarations

AB and JPG are Explicyte employee.

## List of abbreviations

CP: cyclophosphamide
HR: hormone receptor
IFN: interferon
OV: oncolytic viruses
