# Supplementary material for: Phase 2 trial of intravenous oncolytic virus JX-594 combined with low-dose cyclophosphamide in patients with advanced breast cancer
Source: Exp Hematol Oncol. 2022 Dec 6;11:104. doi: 10.1186/s40164-022-00338-2 (PMC9724410; doi:10.1186/s40164-022-00338-2)
Supplement: Supplementary file 1 — Supplementary Material 1: Supplementary Methods and Results [file 40164_2022_338_MOESM1_ESM.docx]

**SUPPLEMENTARY METHODS**

**Patients**

Patients had to be aged 18 years or older and have histologically confirmed metastatic with documented disease progression [as per Response Evaluation Criteria in Solid Tumours (RECIST 1.1)]1 within 6 months prior to entry into the study. Main eligibility criteria included: Age ≥ 18 years ; ECOG ≤ 1; Life expectancy > 3 months ; Measurable disease according to RECIST v1.1 outside any previously irradiated field; At least three weeks since last chemotherapy, immunotherapy or any other pharmacological treatment and/or radiotherapy; Adequate hematological, renal, metabolic and hepatic functions; women of childbearing potential must have a negative serum pregnancy test before study entry. Both women and men must agree to use a medically acceptable method of contraception throughout the treatment period and for six months after discontinuation of treatment; patients receiving any substances that are inhibitors or inducers of CYP450 2B6 are ineligible. All patients signed written informed consent prior to any study specific procedure.

**Study design and treatment**

This was a single-arm, phase 2, clinical trial based on Simon’s two-stage design and was conducted in accordance with the Declaration of Helsinki and Good Clinical Practices. Patients received 50 mg of CP orally b.i.d. one week on and one week off and JX-594 1.10^9^ every two weeks for the first 3 infusions and then every 3 weeks, (21-day cycle). Patients discontinued treatment if one of the following occurred: the patient made the decision to withdraw or there was unacceptable toxicity, disease progression as per RECIST 1.1, undercurrent illness, or changes in the patient’s condition preventing further treatment by the judgement of the investigator. Response assessment was carried out every 6 weeks. The response was determined per RECIST 1.13 guidelines after blinded central imaging review. Toxicities were assessed continuously per Common Terminology Criteria for Adverse Events 4.0.

**Correlative studies**

Plasma samples were collected at baseline and at several time points during the study. Proteomic analysis was performed by using the Olink technology as previously described (see reference below)

**Statistical analysis**

The primary endpoint was objective response within 6 months of treatment onset as per RECIST 1.1. Simon’s two-stage design7 was used. To distinguish a favourable OR rate of 20% from a null rate of 5%, with 80% power and 5% type I error, 29 eligible and assessable patients were required. Following the inclusion of the first 10 assessable patients per cohort, accrual could continue for a total of 29 patients cohort if at least 1 objective response was observed. At the end of recruitment, at least 4 objective response were needed to conclude that the regimen had a meaningful effect. Secondary endpoints included the best overall response per RECIST 1.1, 1-year progression-free survival (PFS), 1-year overall survival (OS), safety, and correlations with the plasma profiling. PFS was defined as the time from the start of treatment to the time of progression or death (from any cause). OS was defined as the time from the start of treatment to death (from any cause) or the last patient contact. Patients who were alive and progression-free were censored at the date of last follow-up. All enrolled patients who received at least one dose of one of the investigational drugs were eligible for safety analyses. To be assessed for the primary efficacy endpoint, a subject had to meet the eligibility criteria and receive at least one dose of CP and one infusion of PEXA-VEC. Descriptive statistics were used to characterize patients at study entry and to report toxicities.

**Reference**:

Loriot Y, Marabelle A, Guégan JP, Danlos FX, Besse B, Chaput N, Massard C, Planchard D, Robert C, Even C, Khettab M, Tselikas L, Friboulet L, André F, Nafia I, Le Loarer F, Soria JC, Bessede A, Italiano A. Plasma proteomics identifies leukemia inhibitory factor (LIF) as a novel predictive biomarker of immune-checkpoint blockade resistance. Ann Oncol. 2021 Nov;32(11):1381-1390.

**SUPPLEMENTARY TABLES**

**Supplementary Table 1: Number of patients presenting at least one adverse event related to Cyclophosphamide and/or JX-594**

| **Maximum intensity** | | | | | | |
| --- | --- | --- | --- | --- | --- | --- |
|  | **Grade 1** | | **Grade 2** | | **Grade 3** | |
|  | **n** | **%** | **n** | **%** | **n** | **%** |
| Anemia | 2 | 20.0 | 1 | 10.0 | . | . |
| Diarrhea | . | . | 1 | 10.0 | . | . |
| Dry mouth | 1 | 10.0 | . | . | . | . |
| Mucositis oral | . | . | 1 | 10.0 | . | . |
| Nausea | 2 | 20.0 | 1 | 10.0 | . | . |
| Vomiting | 4 | 40.0 | 2 | 20.0 | . | . |
| Chills | 1 | 10.0 | . | . | . | . |
| Fatigue | 4 | 40.0 | 1 | 10.0 | . | . |
| Fever | 6 | 60.0 | 7 | 70.0 | 1 | 10.0 |
| Pain | . | . | 1 | 10.0 | . | . |
| Skin rash | 3 | 30.0 | 1 | 10.0 | . | . |
| Alanine aminotransferase increased | 1 | 10.0 | . | . | . | . |
| Alkaline phosphatase increased | . | . | 1 | 10.0 | . | . |
| Aspartate aminotransferase increased | 1 | 10.0 | . | . | . | . |
| GGT increased | . | . | . | . | 1 | 10.0 |
| Lymphopenia | 3 | 30.0 | 1 | 10.0 | 1 | 10.0 |
| Weight loss | 1 | 10.0 | . | . | . | . |
| Dysgeusia | 1 | 10.0 | . | . | . | . |
| Headache | 1 | 10.0 | . | . | . | . |
| Dry skin | 1 | 10.0 | . | . | . | . |
| Hypertension | . | . | . | . | 1 | 10.0 |

|  |  |  |  |  |  |  |
| --- | --- | --- | --- | --- | --- | --- |

**SUPPLEMENTARY FIGURES**

**
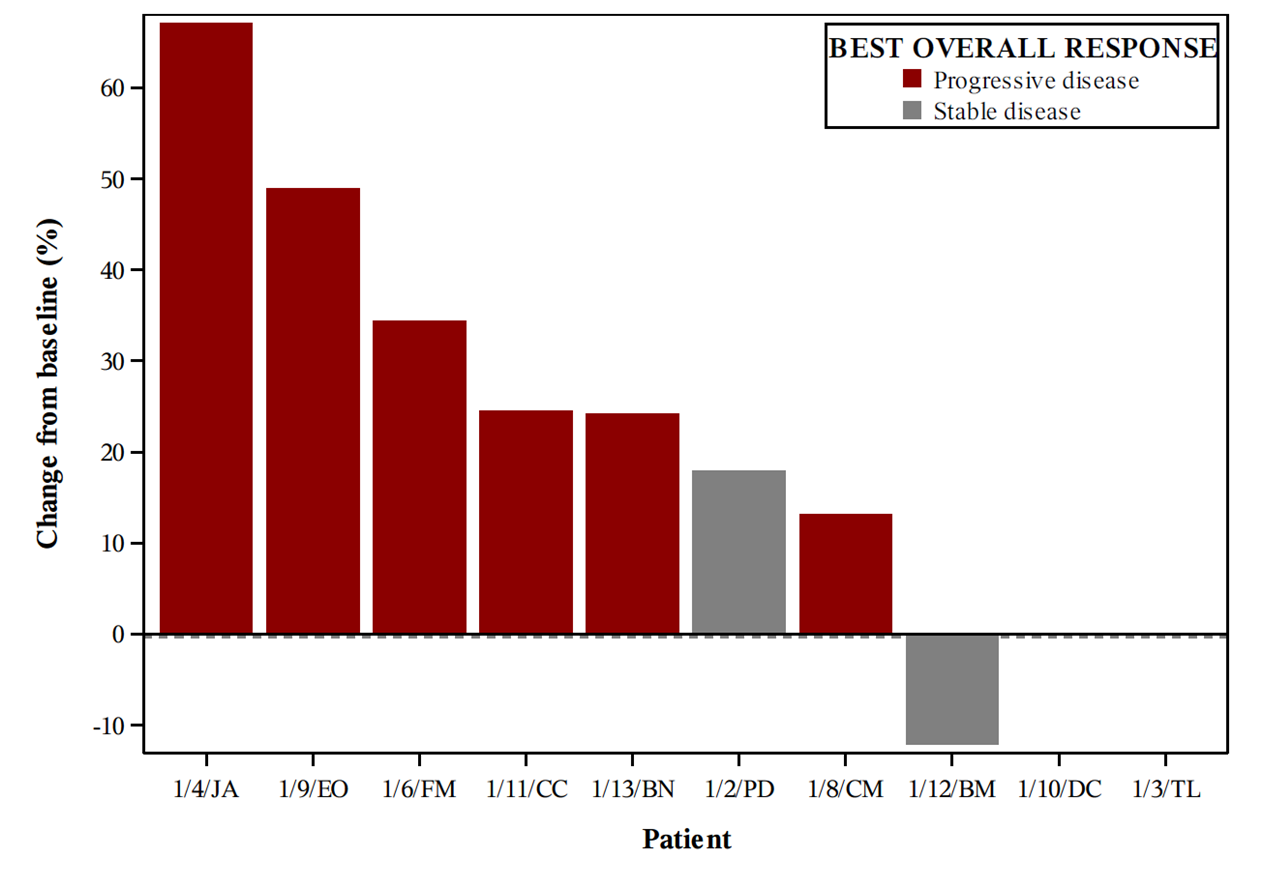
**

**Supplementary Figure 1.** Waterfall plot: patients are ordered according to the change from baseline (%) in the size of target lesions observed at the timing of the best overall response under treatment. Colour code represents the best overall response under treatment. Patient 1/10 had no evaluable target lesions at tumoral evaluation but progression in non-target lesions. Patient 1/3 dead before first evaluation due to progression.


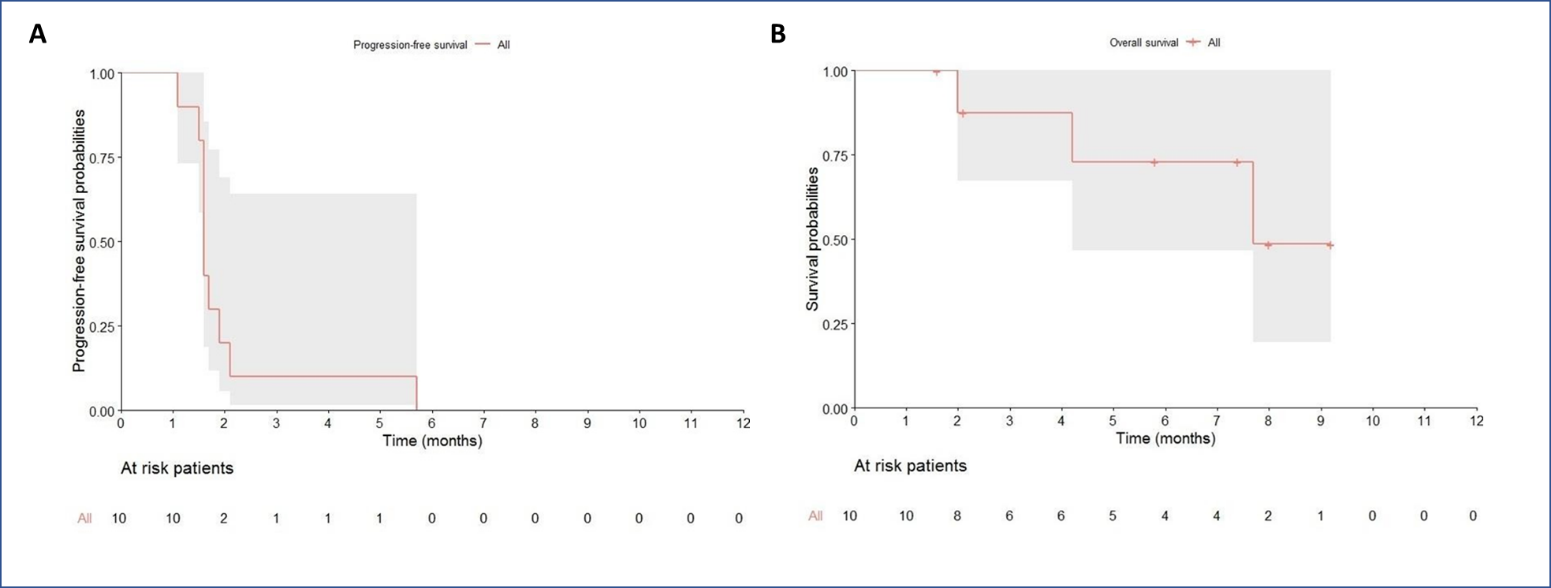


**Supplementary Figure 2.** Kaplan-Meier curves of progression-free (A) and overall survival (B) of patients (n=10) treated with low-dose cyclophosphamide and intravenous PEXA-VEC
